# Probabilistic atlas for the language network based on precision fMRI data from >800 individuals

**DOI:** 10.1038/s41597-022-01645-3

**Published:** 2022-08-29

**Authors:** Benjamin Lipkin, Greta Tuckute, Josef Affourtit, Hannah Small, Zachary Mineroff, Hope Kean, Olessia Jouravlev, Lara Rakocevic, Brianna Pritchett, Matthew Siegelman, Caitlyn Hoeflin, Alvincé Pongos, Idan A. Blank, Melissa Kline Struhl, Anna Ivanova, Steven Shannon, Aalok Sathe, Malte Hoffmann, Alfonso Nieto-Castañón, Evelina Fedorenko

**Affiliations:** 1 Department of Brain and Cognitive Sciences, Massachusetts Institute of Technology, Cambridge, MA USA; 2 McGovern Institute for Brain Research, Massachusetts Institute of Technology, Cambridge, MA USA; 3 Department of Cognitive Science, Johns Hopkins University, Baltimore, MD USA; 4 Human-computer Interaction Institute, Carnegie Mellon University, Pittsburgh, PA USA; 5 Department of Cognitive Science, Carleton University, Ottawa, ON Canada; 6 Department of Psychology, Columbia University, New York, NY USA; 7 Harris School of Public Policy, University of Chicago, Chicago, IL USA; 8 Department of Bioengineering, University of California, Berkeley, CA USA; 9 Department of Psychology, University of California, Los Angeles, CA USA; 10 Athinoula A. Martinos Center for Biomedical Imaging, Massachusetts General Hospital, Cambridge, MA USA; 11 Department of Speech, Language, and Hearing Sciences, Boston University, Boston, MA USA; 12 Department of Speech, Hearing, Bioscience, and Technology, Harvard University, Cambridge, MA USA

**Keywords:** Language, Cognitive neuroscience

## Abstract

Two analytic traditions characterize fMRI language research. One relies on averaging activations across individuals. This approach has limitations: because of inter-individual variability in the locations of language areas, any given voxel/vertex in a common brain space is part of the language network in some individuals but in others, may belong to a distinct network. An alternative approach relies on identifying language areas in each individual using a functional ‘localizer’. Because of its greater sensitivity, functional resolution, and interpretability, functional localization is gaining popularity, but it is not always feasible, and cannot be applied retroactively to past studies. To bridge these disjoint approaches, we created a *probabilistic functional atlas* using fMRI data for an extensively validated language localizer in 806 individuals. This atlas enables estimating the probability that any given location in a common space belongs to the language network, and thus can help interpret group-level activation peaks and lesion locations, or select voxels/electrodes for analysis. More meaningful comparisons of findings across studies should increase robustness and replicability in language research.

## Abstract (summary)

**Table:** 

Measurement(s)	Brain activity measurement
Technology Type(s)	fMRI
Sample Characteristic - Organism	Homo sapiens

## Background & Summary

fMRI is an invaluable non-invasive tool for illuminating the brain’s architecture, especially for human-unique abilities like language. A common analytic approach in fMRI language studies is to average activation maps voxel-wise in a common brain space and perform statistical inference across individuals in each voxel. However, because of the well-established inter-individual variability in the locations of functional areas in the association cortex^1,2^, activations do not align well across individuals, leading to low sensitivity and functional resolution^3^. Further, the results of group-averaging analyses are generally interpreted through reverse inference from anatomical locations to function^4,5^, but because of the variability mentioned above, combined with the functional heterogeneity of the association cortex, locations in a common brain space cannot be meaningfully linked to function (see^6^ for a discussion of this issue for ‘Broca’s area’).

An alternative analytic approach, which circumvents voxel-wise brain averaging, is known as ‘functional localization’^3,7^. In this approach, a brain region or network that supports a mental process of interest is identified with a functional contrast in each individual and then its responses to some new critical condition(s) are examined. This approach yields greater sensitivity, functional resolution, and interpretability, and has been successful across many domains of perception and cognition, including language. As a result, many research groups are now moving away from group-averaging analyses toward individual-subject analyses^4,8^.

However, functional localization is not always feasible. Further, although studies that rely on functional localization can be straightforwardly compared to each other, it is at present unclear how to relate the results from such studies to group-averaging fMRI studies, or other studies that rely on brain averaging (e.g., studies that use voxel-based morphometry (VBM) or voxel-based lesion-symptom mapping (VLSM) in patient work^9^). To help bridge the gap between these two analytic traditions in language research, we created a ***probabilistic functional atlas of the language network (‘Language Atlas’ or LanA)*** by overlaying 806 individual activation maps for a robust at the individual-subject level and extensively validated language ‘localizer’^10,11^.

The language localizer relies on a contrast between the processing of sentences and a linguistically/acoustically degraded control condition and is robust to changes in materials, modality of presentation, and task (see Methods). This localizer identifies the left-lateralized fronto-temporal language network (e.g.^12–14^) that selectively^6,15^ supports high-level language comprehension and production^16–18^, including the processing of word meanings and combinatorial syntactic/semantic processing^19–21^. By design, this contrast excludes lower-level perceptual^22–25^ and speech-articulatory^26–28^ processes, as well as discourse-level comprehension^29–32^. Further, a network that closely corresponds to this functional contrast emerges from task-free resting state data^33^. (Many researchers have postulated functional dissociations among the different brain regions that comprise the language network (e.g.^12–14,34–36^). However, the empirical landscape remains complex and ridden with controversy, and the evidence is now overwhelming that—even if dissociations exist within the network— all the language regions are strongly synchronized in their activity^33,37,38^, suggesting that they form a functionally integrated system).

**Fig. 1:**
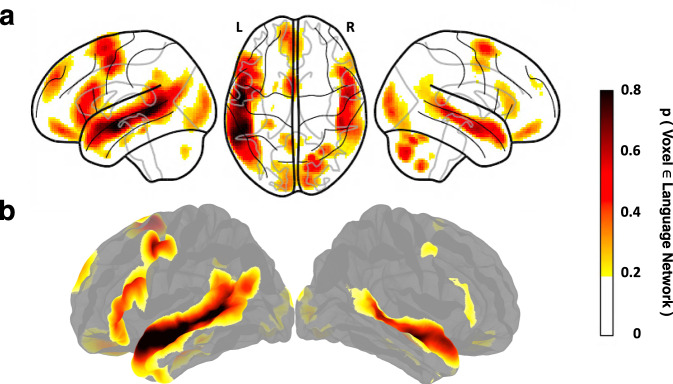
Language atlas topography. Probabilistic functional atlas for the *language* > *control* contrast based on overlaid individual binarized activation maps (where in each map, the top 10% of voxels are selected, as described in the text). (**a**) SPM-analyzed volume data in the MNI template space (based on 806 individual maps). (**b**) FreeSurfer-analyzed surface data in the FSaverage template space (based on 804 individual maps). In both figures, the color scale reflects the proportion of participants for whom that voxel/vertex belongs to the top 10% of *language* > *control* voxels/vertices (threholded at p = 0.2 for visualization purposes).

LanA allows one to estimate for any location in a common brain space the probability that it falls within the language network. In this way, this atlas can provide a common reference frame and help interpret (a) group-level activation peaks from past and future fMRI studies, or results of meta-analyses of such peaks^39^, (b) lesion locations in individual brains^40^ or lesion overlap loci in VBM/VLSM analyses, and (c) electrode locations in ECoG/SEEG investigations or locations of source-localized activity in MEG studies. Furthermore, LanA (d) can help select language-selective units (voxels, electrodes, MEG channels, even single cells) for analysis in existing datasets, including in studies that aim to relate human neural representations to those from artificial neural network language models^41–46^, (e) can be related voxel-by-voxel to any whole-brain data^47^, including structural data, gene expression data^48^, or receptor density data^49^, in order to ask whether/how these features correlate with the language network’s topography, and (f) can help select patches in post-mortem brains for cellular analyses to maximize the chances of examining language cortex. Finally, LanA (g) can help guide/constrain functional mapping during brain surgery when fMRI is not possible, although, of course, no clinical decisions should be made based on LanA alone. We make the atlas available for two most commonly used brain templates (Fig. 1): a volume-based template (MNI IXI549Space; SPM12^50^) and a surface-based template (fsaverage; FreeSurfer^51^). The use of these common data formats will allow for easy interfacing with existing open data repositories such as NeuroVault^52^ and ENIGMA^53^. We emphasize that LanA is not a replacement for localizers: when possible, a language localizer task should be performed^54^. As we show in SI-1, the effect sizes obtained from group-level regions of interest (ROIs) based on LanA, or from commonly used Glasser parcels^55^ are underestimated relative to individually defined language functional ROIs.

We also release (i) individual activation maps (in the MNI and FSaverage spaces), along with demographic data, and (ii) individual-level neural markers (based on the volumetric analyses), including effect sizes, voxel count (activation extent), and stability of activation across runs. The neural marker data can be used as normative distributions, based on neurotypical relatively young adults, against which any new population (e.g., children or individuals with developmental or acquired brain disorders) can be evaluated.

## Methods

### Participants

A total of 806 neurotypical adults (477, ~59%, female), aged 19 to 75 (441, ~55%, aged 19–29; 310, ~38%, aged 30–39; 55, ~7%, aged 40+), participated for payment between September 2007 and June 2021, as summarized in Table 1. All participants had normal or corrected-to-normal vision, and no history of neurological, developmental, or language impairments. Handedness information was collected for 758 (~94%) of the 806 participants. Of those, 707 participants (~93%) were right-handed, as determined by the Edinburgh handedness inventory^56^ or self-report, 38 (~5%) were left-handed, and 13 (~2%) – ambidextrous. (The participants for whom handedness is missing in the database are most likely right-handed because most of them were tested during the earlier years of data collection when right-handedness was one of the requirements for participation.) Of the 806 participants, 629 (~78%) were native speakers of English, and the remaining 177 (~22%) – native speakers of another language and proficient speakers of English (see^38^ for evidence that the topography of language responses for a language that an individual is proficient in is similar to that of their native language, and see SI-2 for a comparison between the atlas generated using the 629 native English speakers vs. the 177 proficient non-native speakers). Given this demographic distribution, this atlas represents certain populations better than others, and these biases should be taken into account when the data are interpreted, including in comparison to other populations.

**Table 1:** Participant demographics.

Age	30.23 ± 7.08	Years
Gender	40.57%	Male
	59.18%	Female
Handedness	87.72%	Right-Handed
	4.71%	Left-Handed
	1.61%	Ambidextrous
	5.96%	No Handedness Info
Native English Speaker Status	78.04%	Native Speakers of English
	21.96%	Proficient Speakers of English

Summary demographics of the 806 participants included in the atlas.

Each participant completed a language localizer task^10^ as part of one of the studies in the Fedorenko lab. Each scanning session lasted between 1 and 2 hours and included a variety of additional tasks. All participants gave informed written consent in accordance with the requirements of the MIT’s Committee on the Use of Humans as Experimental Subjects (COUHES).

### Participant and session selection

The 806 scanning sessions above (one session per participant) were selected from a total of 1,065 sessions across 819 participants that were available in the Fedorenko Lab database as of June 2021. The goal was to include as many participants as possible and, for the 163 participants who performed a language localizer in multiple sessions, to select a single session with high-quality data. To assess data quality, we examined the stability of the activation topography for the language localizer contrast (see Language localizer paradigm) across runs. This analysis was performed on the data preprocessed and analyzed in the volume (i.e., SPM-based analyses; see SPM preprocessing and analysis pipeline). For 1,062 of the 1,065 sessions, we calculated voxel-wise spatial correlations in activation magnitudes the *language* > *control* contrast (see Language localizer paradigm) between the odd-numbered and even-numbered runs (the three remaining sessions consisted of a single run and were evaluated by visual inspection of the contrast maps). The correlation values were calculated within the language ‘parcels’—masks that denote typical locations of language areas. These masks (available at^54^ http://evlab.mit.edu/funcloc) were derived from a probabilistic language atlas based on 220 participants (a subset of the participants in the current set of 806) and have been used in much past work^57–63^. Six masks (three in the frontal cortex and three in the temporal and parietal cortex) were derived from the probabilistic atlas in the left hemisphere and mirror-projected onto the right hemisphere. For each session, the correlation values were averaged across the twelve parcels, leading to a single value per session. This spatial correlation measure quantifies the stability of the activation landscape and is an objective proxy for data quality; it is affected by factors like head motion or sleepiness, but does not require subjective visual inspection of contrast maps (see SI-4 for evidence that this measure works similarly when considering all voxels vs. only voxels with positive values for the contrast of interest, suggesting that the values are not driven by the difference between responsive and non-responsive voxels). Sessions where the spatial correlation value was negative (n = 23; ~2%) were excluded, leaving 1,042 sessions across 806 participants. For the 163 participants with more than one session, we selected the session with the highest spatial correlation value for inclusion in the atlas (see^11^ for evidence of the stability of spatial correlation values across sessions: i.e., if a participant shows a high spatial correlation in one session, they are likely to show a high spatial correlation in another session; unpublished data replicates this result across a larger population and multiple functionally distinct networks). Following this data selection procedure, the Fisher-transformed spatial correlations of the participants’ *language* > *control* contrast were *r* = 0.98 and *r* = 0.57 for the left and right hemispheres, respectively (see^11^ for similar values on a subset (n = 150) of these participants).

### Language localizer paradigm

Across the 806 participants, ten language localizer versions were used, as summarized in Table 2. In each version, a sentence comprehension condition was contrasted with a linguistically or acoustically degraded control condition. Visual (reading) and auditory (listening) contrasts have been previously established to engage the same fronto-temporal language network^10,38,64,65^. Activity in this network has further been shown to not depend on task or materials^10^ and to show robust effects across typologically diverse languages^38^. Furthermore, this network can be recovered from naturalistic task-free (resting state) data based on patterns of BOLD signal fluctuations over time^33,37^, and corresponds nearly perfectly to the network based on the *sentences* > *nonwords* contrast^10^. As a result, we pooled data from across the different localizer versions in the current study (see SI-2 for evidence that an atlas defined on only ***Localizer Version A***, used in the majority of participants, is nearly identical to an atlas that leverages data from all other versions, and SI-3 for a supplementary analysis showing robust *language* > *control* effects across all ten versions).

**Table 2:** Language localizer versions.

Version	A	B	C	D	E	F	G	H	I	J
Number of participants	624	67	60	15	17	6	4	8	4	1
Task type	BP	MP	N/MP	MP	MP	BP	MP	N	MP	N
Words/Nonwords per trial	12	8	12	12	8	12	8	Variable	8	Variable
Modality	Visual	Visual	Visual	Visual	Visual	Visual	Visual	Auditory	Auditory	Auditory
Trial duration (ms)	6000	4800	6000	6000	4800	4800	4800	18000	6000	12000
Trial-initial Fixation	100	300	300	300	300	600	300			
Stimulus	5400 (450/word)	2800 (350/word)	4200 (350/word)	4200 (350/word)	2800 (350/word)	4200 (350/word)	2800 (350/word)		3300–4300	
Button icon/Memory probe	400	1350	0 or 1000	1000	1350		1350		≤1000	
Trial-final Fixation	100	350	500 or 1500	500	350		350		Until 6000	
Trials per block	3	5	3	3	5	5	5	1	4	1
Block duration (s)	18	24	18	18	24	24	24	18	24	12
Blocks per condition per run	8	4	8	6	4	4	8	8	4	16
**Conditions**	**Sentences, Nonwords**	**Sentences, Wordlists, Nonwords**	**Sentences, Nonwords**	**Sentences, Wordlists, Nonwords**	**Sentences, Wordlists, Jabberwocky, Nonwords**	**Sentences, Wordlists, Jabberwocky, Nonwords**	**Sentences, Nonwords**	**Intact, Degraded**	**Sentences, Wordlists, Jabberwocky, Nonwords**	**Sentences, Nonwords**
Fixation block duration (s)	14	16	18	18	16	25	16	14	16	16
Fixation blocks per run	5	3	5	4	5	4	5	5	5	5
Total run time (s)	358	336	378	396	464	504	464	358	464	464
Number of runs	2	2–5	2	2–3	6–8	2–8	2	2	4	2

Timing parameters for each version of the language localizer task. Under task type, the options are defined as follows: BP = Button Press, MP = Memory Probe, N = No Task. (For the Memory Probe task, the correct probes were approximately equally likely to come from early, middle, and late parts of the string).

The vast majority of participants (624, ~77.4%) performed ***Localizer version A*** – a reading version, where sentences and nonword strings are presented one word/nonword at a time at the rate of 450 ms per word/nonword, in a blocked design (with 3 sentences/nonword strings in each 18 s block). Participants were instructed to read attentively and to press a button at the end of each trial, when a picture of a finger pressing a button appeared on the screen. The experiment consisted of two ~6-minute-long runs, for a total of 16 blocks for each of the two conditions. The presentation script and stimuli for this localizer version can be downloaded at^54^ http://evlab.mit.edu/funcloc/ (for the stimuli used in the other localizer versions, contact EF). ***Localizer versions B-G*** (performed by 169 participants, ~21.0%) also used visual presentation, and ***Localizer versions H-J*** (performed by 13 participants, ~1.6%) used auditory presentation. Details of the similarities and differences in trial structure, timing, and other experimental parameters across versions are summarized in Table 2.

### fMRI data acquisition

Structural and functional data were collected on the whole-body, 3 Tesla, Siemens Trio scanner with a 12-channel (G1; n = 18) or 32-channel (G2; n = 788) head coil, at the Athinoula A. Martinos Imaging Center at the McGovern Institute for Brain Research at MIT. T1-weighted structural images were collected in 176 sagittal slices with 1 mm isotropic voxels (TR = 2,530 ms, TE = 3.48 ms). Functional, blood oxygenation level dependent (BOLD), data were acquired using an EPI sequence (with a 90 degree flip angle and using GRAPPA with an acceleration factor of 2), with the following acquisition parameters: 33 (G1) or 31 (G2) 4 mm thick near-axial slices acquired in the interleaved order (with 10% distance factor), 3.0 mm × 3.0 mm (G1) or 2.1 mm × 2.1 mm (G2) in-plane resolution, FoV in the phase encoding (A ≫ P) direction 192 mm (G1) or 200 mm (G2) and matrix size 64 mm × 64 mm (G1) or 96 mm × 96 mm (G2), TR = 2,000 ms and TE = 30 ms. Prospective acquisition correction^66^ was used to adjust the positions of the gradients based on the participant’s motion from the previous TR. The first 10 s of each run were excluded to allow for steady state magnetization.

### SPM preprocessing and analysis pipeline

#### Preprocessing

**Fig. 2:**
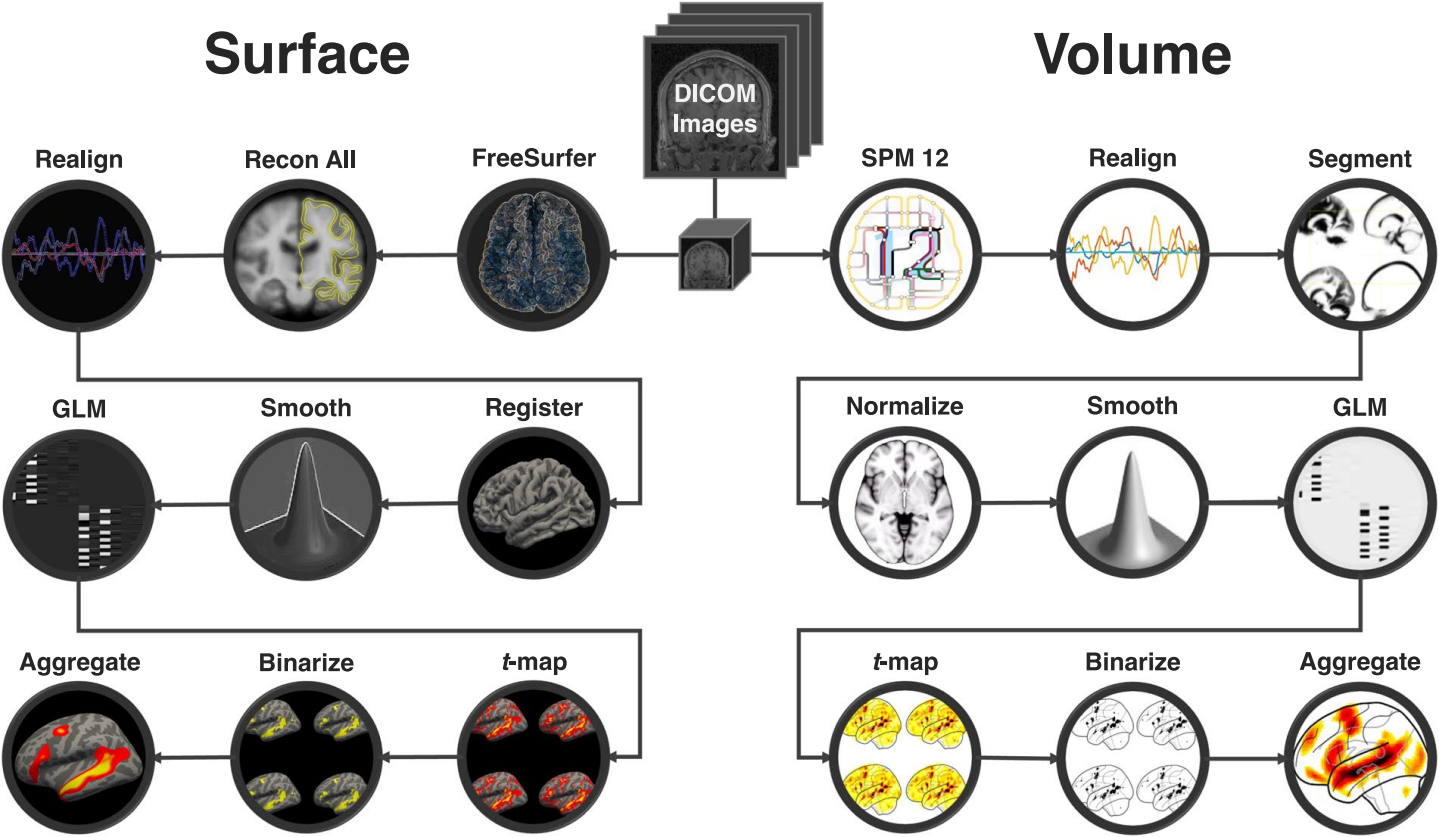
Data processing flowchart. Overview of the SPM and FreeSurfer preprocessing and analysis pipelines. Raw dicom images were converted to NIfTI format, motion-corrected, mapped to a common space and smoothed during preprocessing. Each session was then modeled, *t*-maps were extracted and thresholded, and all sessions were aggregated to create the probabilistic atlas.

For the SPM analyses (Fig. 2 [**volume]**), fMRI data were analyzed using SPM12 (release 7487), CONN EvLab module (release 19b), and custom MATLAB scripts. Each participant’s functional and structural data were converted from DICOM to NIfTI format. All functional scans were coregistered and resampled using B-spline interpolation to the first scan of the first session (Friston *et al*.^67^). Potential outlier scans were identified from the resulting subject-motion estimates as well as from BOLD signal indicators using default thresholds in CONN preprocessing pipeline (5 standard deviations above the mean in global BOLD signal change, or framewise displacement values above 0.9 mm; Nieto-Castañón^68^). Functional and structural data were independently normalized into a common space (the MontrealNeurological Institute [MNI] template; IXI549Space) using SPM12 unified segmentation and normalization procedure (Ashburner & Friston^69^) with a reference functional image computed as the mean functional data after realignment across all timepoints omitting outlier scans. The output data were resampled to a common bounding box between MNI-space coordinates (−90, −126, −72) and (90, 90, 108), using 2 mm isotropic voxels and 4th order spline interpolation for the functional data, and 1 mm isotropic voxels and trilinear interpolation for the structural data. Last, the functional data were smoothed spatially using spatial convolution with a 4 mm FWHM Gaussian kernel.

#### First-level analysis

Effects were estimated using a General Linear Model (GLM) in which each experimental condition was modeled with a boxcar function convolved with the canonical hemodynamic response function (HRF) (fixation was modeled implicitly, such that all timepoints that did not correspond to one of the conditions were assumed to correspond to a fixation period). Temporal autocorrelations in the BOLD signal timeseries were accounted for by a combination of high-pass filtering with a 128 seconds cutoff, and whitening using an AR(0.2) model (first-order autoregressive model linearized around the coefficient a = 0.2) to approximate the observed covariance of the functional data in the context of Restricted Maximum Likelihood estimation (ReML). In addition to experimental condition effects, the GLM design included first-order temporal derivatives for each condition (included to model variability in the HRF delays), as well as nuisance regressors to control for the effect of slow linear drifts, subject-motion parameters, and potential outlier scans on the BOLD signal.

### FreeSurfer preprocessing and analysis pipeline

For the FreeSurfer analysis (Fig. 2 [**surface**]), fMRI data were analyzed using FreeSurfer v6.0.0. Each participant’s functional and structural data were converted from DICOM to NIfTI format using the default *unpacksdcmdir* parameters. (Two of the 806 participants could not be included in this pipeline because their raw dicom files were lost, leaving 804 participants for this analysis.) The raw data were then sampled onto both hemispheres of the FSaverage surface, motion corrected and registered using the middle time point of each run. The data were then smoothed spatially with a 4 mm FWHM Gaussian filter. For the first-level analyses, effects were estimated using a GLM in which each condition was modeled with a first order polynomial regressor fitting the canonical HRF. The GLM also included nuisance regressors for offline-estimated subject-motion parameters.

### Atlas creation

#### SPM

Using custom code (available at OSF^70^), we computed the overlap of the individual activation maps for the *language* > *control* contrast using the 806 participants analyzed in the SPM12 pipeline (see SI-5 for evidence that the atlas reaches stability at sample sizes much smaller than 806, which suggests that the current sample size is sufficient to be generalizable). In particular, we used whole-brain *t*-maps that were generated by the first-level analysis and that contain a *t*-value for the relevant contrast in each voxel (a post-hoc analysis compared the whole-brain *t*-maps to their corresponding unscaled contrast maps and found strong voxel-wise correlations over the set of 806 participants: *r* = 0.93 ± 0.03; see SI-2 for evidence that atlases generated from *t*-maps vs. contrast maps are highly similar). In each individual map, we selected the 10% of voxels across the brain with the highest *t*-values for the *language* > *control* contrast (average and median *minimum t-*values across participants were 1.73 and 1.62, respectively; average and median *maximum t*-values were 13.8 and 13.7, respectively). These maps were then binarized so that the selected voxels got assigned a value of 1 and the remaining voxels—a value of 0. Finally, these values were averaged across participants in each voxel. The resulting atlas contains in each voxel a value between 0 and 1, which corresponds to the proportion of the 806 participants for whom that voxel falls in the 10% of voxels across the brain with the highest *t*-values for the *language* > *control* contrast. In the left hemisphere, these values range from 0 to 0.82, and in the RH—from 0 to 0.64 (the values are lower in the RH presumably because the majority of the selected voxels fall in the LH: across participants, average and median proportions of selected voxels falling in the LH are 58.3% and 57.8%, respectively). For more details on ROI-level probability values, see SI-6.

#### FreeSurfer

Using custom code (available at OSF^70^), we computed the overlap of the individual activation maps for the *language* > *control* contrast, using the 804 participants analyzed in the FreeSurfer pipeline. The procedure was similar to that used for the SPM-based atlas, except that the selection of the highest *t-*values was performed on the surface vertices. To maintain hemispheric asymmetries, rather than evaluating each hemisphere separately, as is generally common for FreeSurfer analyses, the top 10% of vertices were selected from the vertices pooled across the LH and RH, as for the SPM-based atlas. For this atlas, in the left hemisphere, the proportion values range from 0 to 0.90, and in the RH—from 0 to 0.80 (these values are expectedly higher than those in the SPM-based atlas given the superiority of surface-based inter-individual alignment^71^).

#### General

We chose the top 10% approach over an approach where each individual map is thresholded at a fixed *t*-value (as in^10^) to account for inter-individual variability in the overall strength of BOLD signal responses due to trait or state factors^72^–^75^. However, because differences in the language network size may correspond to differences in linguistic experience or ability^76^, we additionally provide versions of the atlas that are derived from *t*-maps that are thresholded at *p* < 0.001, *p* < 0.01, or *p* < 0.05 (10.17605/OSF.IO/KZWBH^70^). Atlases based on the fixed *t*-value thresholding approach yield topographies that are very similar to the one based on the top 10% approach (see SI-2). The critical difference between these versions of the atlas is in the *interpretation of the overlap values*: whereas, as noted above, in the top 10% approach, the overlap values correspond to the proportion of the 806 participants for whom that voxel falls in the 10% of voxels across the brain with the highest *t*-values for the *language* > *control* contrast, in the atlases based on the fixed-thresholding approach, the overlap values correspond to the proportion of the 806 participants for whom that voxel is significant for the *language* > *control* contrast at the relevant threshold.

Note that in addition to the classic left frontal and left temporal areas (and their right-hemispheric homotopes), several other areas emerge in the atlas, including in the right cerebellum and in the visual cortex. These less canonical areas have been reported in past language studies (e.g.^77^,^78^), but we acknowledge that in general, these have not been as thoroughly functionally characterized as the core frontal and temporal areas, and may in future work be shown not to be selective and/or critical for language function.

Finally, one might ask: how similar is the topography of a probabilistic functional atlas to that of a random-effects group map for the same data. Of course, these are expected to be correlated given that voxels which are task-responsive in a greater portion of the participant population (i.e., have higher probability overlap values in the atlas) are likely to yield higher *t*-values in the voxel-wise *t*-tests (see SI-7 for this comparison for LanA). The critical advantage of the probabilistic functional atlas like LanA over a random-effects map is the straightforward interpretation of the voxel values that it affords, in terms of the probability that the voxel belongs to the relevant functional area/network (the language network in this case). Such information cannot be inferred from *t*-values in a random-effect map without additional assumptions/mapping functions.

### Neural markers

In addition to the population-level atlases, we also provide a set of individual-level neural markers (based on the volumetric SPM analyses) for the language network in each participant. These neural markers include: effect size, voxel count, and spatial correlation (additional information on these markers provided below). All of these markers have all been shown to be reliable within individuals over time, including across scanning sessions^11^. We provide each of these measures for each of the ROIs constrained by the previously defined language ‘parcels’ (available at^54^ http://evlab.mit.edu/funcloc), which include in each hemisphere three frontal parcels (inferior frontal gyrus [IFG], its orbital portion [IFGorb], and middle frontal gyrus [MFG]) and three temporal/parietal ones (anterior temporal [AntTemp], posterior temporal [PostTemp], and angular gyrus [AngG]), for a total of 12 parcels. Of the 806 participants included in the atlas, only 803 completed the 2 or more runs, as needed to calculate the effect size and spatial correlation markers; for the remaining 3 participants, only voxel count is provided.

Effect size was operationalized as the magnitude (% BOLD signal change) of the critical *language* > *control* contrast. Within each parcel, we defined—for each participant—a functional ROI (fROI) by selecting 10% of the mask’s total voxels with the highest *t-values* for the *language* > *control* contrast using all but one run of the data. We then extracted from the left-out run the responses to the language and control conditions and computed the *language* > *control* difference. This procedure was repeated across all run partitions. This across-runs cross-validation procedure^3^ ensures independence between the data used to define the fROIs and estimate their responses^79^. In the final step, the estimates were averaged across the cross-validation folds to derive a single value per participant per fROI. Voxel count (activation extent) was defined as the number of significant voxels for the critical *language* > *control* contrast at a fixed statistical threshold (*p* < 0.001 uncorrected threshold). Spatial correlation (stability of the activation landscape) was defined—for the voxels falling within the language parcels—as the Fisher-transformed Pearson correlation coefficient between the voxel responses for the *language* > *control* contrast across odd- and even-numbered runs. As noted above, for all three measures, we provide 14 values per participant: one for each of the 12 ROIs (6 in each hemisphere), and two additional per-hemisphere values (averaging across the 6 ROIs in each hemisphere). See Table 3 for a summary of these neural markers within the atlas population. Additional measures can be computed based on the measures we provide (e.g., lateralization can be computed from the voxel counts^80^), and other measures can be extracted from the whole-brain activation maps (see Data records).

**Table 3:** Neural marker distributions.

Neural Markers	Minimum	0.25	Median	0.75	Max
LH Effect Size	−0.28	0.88	1.28	1.65	3.68
RH Effect Size	−0.40	0.20	0.43	0.73	2.51
LH Voxel Count	0	1197	1908	2594	4887
RH Voxel Count	0	196	495	1044	4473
LH Spatial Correlation	−0.01	0.74	1.00	1.23	1.90
RH Spatial Correlation	−0.29	0.33	0.57	0.78	1.75

Summary of the neural markers for the *language* > *control* contrast of the 803 participants included in the atlas for whom we have 2 or more runs. Effect sizes reflect the % BOLD signal change for the *language* > *control* contrast in the language fROIs (estimated using across-runs cross-validation, as described in the text). Voxel counts reflect the number of significant voxels for the critical *language* > *control* contrast at a fixed statistical threshold (*p* < 0.001 uncorrected) within the language parcel boundaries (see text for details; Neural markers). Spatial correlation is defined as the Fisher-transformed Pearson correlation coefficient between the voxel responses for the *language* > *control* contrast across odd- and even-numbered runs within the language parcel boundaries. LH = Left Hemisphere; RH = Right Hemisphere. The columns show the values that correspond to the minimum value, the value at the 25^th^ percentile of the population distribution, the median, the value at the 75^th^ percentile, and the maximum value.

These different measures can be explored with respect to each other, or to the demographic variables (but see^81^ for a discussion about the prevalence of underpowered brain-behavior individual differences studies). These measures can also serve as normative distributions against which any new population can be evaluated, including children or individuals with developmental and acquired brain disorders, or otherwise atypical brains^82^.

## Data Records

The full dataset, including the SPM and FreeSurfer atlases are available for download^83^ (10.6084/m9.figshare.20425209). Along with the atlases, we make available i) individual contrast and significance maps (for both the volume-based SPM and the surface-based FreeSurfer pipelines; because we had not obtained consent for raw data release, we cannot make publicly available the raw dicom/NIfTI images), and ii) a dataset of individual neural markers.

The complete dataset can additionally be accessed at http://evlabwebapps.mit.edu/langatlas/ via the prepackaged download links. The ‘Download SPM Atlas’ and ‘Download FS Atlas’ links provide a copy of the language atlas in their respective formats. The SPM atlas is a single volumetric NIfTI file, whereas the FS atlas is comprised of two overlay NIfTI files, one for each hemisphere. Under ‘Download All SPM Data’ and ‘Download All FS Data’, each of the individual participant’s data can be downloaded. In particular, for each of the 806 participants (804 for FS), we provide a ‘Demograhics_&_Summary.txt’ file, which contains relevant information as in Tables 2 and 3, the individual contrast and significance maps, and a visualization of their individual activation profile in the selected template space.

As well as allowing the user to download the data, the LanA website offers opportunities for online exploration and the retrieval of relevant subsets of data. In particular, individual activation maps can be explored under the ‘Explore Activation Maps’ tab, and relevant neural markers can be explored under the ‘Explore Neural Markers’ tab. In addition, data can be filtered by demographic variables including, age, gender, handedness, native English speaker status, language network lateralization, etc., and these subsets can be downloaded, or their maps/neural markers can be explored. This flexible tool allows individual users to access relevant data for their needs without the requirement for offline filtering.

Finally, at^54^, we provide a version of the language localizer experiment (*Localizer Version A*, which is used for the majority of participants) for download.

## Technical Validation

The individual participants’ data quality check was performed as described in the Participants and session selection section.

Individual localizer versions were evaluated to confirm they each elicited a strong *language* > *control* effect, as described in SI-3.

The atlas creation process was evaluated with respect to several hyperparameter choices, and the atlas remained robust to each decision, including the inclusion of non-native but proficient English speakers, different localizer versions, the use of whole-brain maps based on *t-*values vs. contrast values, and definition of the language system as the top 10% of *language* > *control* voxels vs. as *language* > *control* voxels that pass a specific significance threshold. We summarize the (minimal) impact of all these choices in SI-2.

Finally, in SI-1, we demonstrate that group-level ROIs defined based on the highest-overlap voxels in LanA outperform commonly used Glasser ROIs derived from multi-modal Human Connectome Project data^55^ in effect size estimation. The latter grossly underestimate effect sizes, especially for the frontal language areas. Of course, as expected^3^, individual-level language fROIs are still the best for accurately estimating effect sizes, and these outperform the group-based LanA fROIs, but in cases where individual localization may not be possible (e.g., in retroactively re-analyzing past studies), LanA-based group ROIs are recommended, as they fare substantially better than Glasser ROIs.

## Usage Notes

The data records presented in this paper, including materials for download and exploration at the http://evlabwebapps.mit.edu are available for free and fair use to individual and academic researchers, institutions, and entities provided that this work is appropriately referenced. Although this atlas has potential for clinical applications, the authors assume no responsibility for the use or misuse of LanA and associated data records in clinical and other settings.

## Supplementary information

Supplementary Information

## Data Availability

Code associated with this manuscript can be found at OSF^70^.

## References

[1] Frost MA, Goebel R (2012). Measuring structural–functional correspondence: Spatial variability of specialised brain regions after macro-anatomical alignment. NeuroImage.

[2] Tahmasebi AM (2012). Is the Link between Anatomical Structure and Function Equally Strong at All Cognitive Levels of Processing?. Cereb. Cortex.

[3] Nieto-Castañón A, Fedorenko E (2012). Subject-specific functional localizers increase sensitivity and functional resolution of multi-subject analyses. NeuroImage.

[4] Fedorenko E (2021). The early origins and the growing popularity of the individual-subject analytic approach in human neuroscience. Curr. Opin. Behav. Sci..

[5] Poldrack RA (2011). Inferring Mental States from Neuroimaging Data: From Reverse Inference to Large-Scale Decoding. Neuron.

[6] Fedorenko E, Blank IA (2020). Broca’s Area Is Not a Natural Kind. Trends Cogn. Sci..

[7] Saxe R (2006). Why and how to study Theory of Mind with fMRI. Brain Res..

[8] Gratton C, Braga RM (2021). Editorial overview: Deep imaging of the individual brain: past, practice, and promise. Curr. Opin. Behav. Sci..

[9] Wilson SM (2017). Lesion-symptom mapping in the study of spoken language understanding. Lang. Cogn. Neurosci..

[10] Fedorenko E, Hsieh P-J, Nieto-Castañón A, Whitfield-Gabrieli S, Kanwisher N (2010). New Method for fMRI Investigations of Language: Defining ROIs Functionally in Individual Subjects. J. Neurophysiol..

[11] Mahowald K, Fedorenko E (2016). Reliable individual-level neural markers of high-level language processing: A necessary precursor for relating neural variability to behavioral and genetic variability. NeuroImage.

[12] Friederici AD (2012). The cortical language circuit: from auditory perception to sentence comprehension. Trends Cogn. Sci..

[13] Price CJ (2012). A review and synthesis of the first 20 years of PET and fMRI studies of heard speech, spoken language and reading. Neuroimage.

[14] Hagoort P, Indefrey P (2014). The Neurobiology of Language Beyond Single Words. Annu. Rev. Neurosci..

[15] Fedorenko E, Behr MK, Kanwisher N (2011). Functional specificity for high-level linguistic processing in the human brain. Proc. Natl. Acad. Sci..

[16] Menenti L, Gierhan SME, Segaert K, Hagoort P (2011). Shared Language: Overlap and Segregation of the Neuronal Infrastructure for Speaking and Listening Revealed by Functional MRI. Psychol. Sci..

[17] Silbert LJ, Honey CJ, Simony E, Poeppel D, Hasson U (2014). Coupled neural systems underlie the production and comprehension of naturalistic narrative speech. Proc. Natl. Acad. Sci..

[18] Hu, J. *et al*. The language network supports both lexical access and sentence generation during language production. 2021.09.10.459596 Preprint at 10.1101/2021.09.10.459596 (2021).

[19] Bautista A, Wilson SM (2016). Neural responses to grammatically and lexically degraded speech. Lang. Cogn. Neurosci..

[20] Fedorenko E, Nieto-Castañon A, Kanwisher N (2012). Lexical and syntactic representations in the brain: An fMRI investigation with multi-voxel pattern analyses. Neuropsychologia.

[21] Fedorenko E, Blank IA, Siegelman M, Mineroff Z (2020). Lack of selectivity for syntax relative to word meanings throughout the language network. Cognition.

[22] Overath T, McDermott JH, Zarate JM, Poeppel D (2015). The cortical analysis of speech-specific temporal structure revealed by responses to sound quilts. Nat. Neurosci..

[23] Norman-Haignere S, Kanwisher NG, McDermott JH (2015). Distinct Cortical Pathways for Music and Speech Revealed by Hypothesis-Free Voxel Decomposition. Neuron.

[24] McCandliss BD, Cohen L, Dehaene S (2003). The visual word form area: expertise for reading in the fusiform gyrus. Trends Cogn. Sci..

[25] Baker CI (2007). Visual word processing and experiential origins of functional selectivity in human extrastriate cortex. Proc. Natl. Acad. Sci..

[26] Bohland JW, Guenther FH (2006). An fMRI investigation of syllable sequence production. NeuroImage.

[27] Basilakos A, Smith KG, Fillmore P, Fridriksson J, Fedorenko E (2018). Functional Characterization of the Human Speech Articulation Network. Cereb. Cortex.

[28] Bouchard KE, Mesgarani N, Johnson K, Chang EF (2013). Functional organization of human sensorimotor cortex for speech articulation. Nature.

[29] Ferstl EC, von Cramon DY (2001). The role of coherence and cohesion in text comprehension: an event-related fMRI study. Cogn. Brain Res..

[30] Lerner Y, Honey CJ, Silbert LJ, Hasson U (2011). Topographic Mapping of a Hierarchy of Temporal Receptive Windows Using a Narrated Story. J. Neurosci..

[31] Blank IA, Fedorenko E (2020). No evidence for differences among language regions in their temporal receptive windows. NeuroImage.

[32] Jacoby N, Fedorenko E (2020). Discourse-level comprehension engages medial frontal Theory of Mind brain regions even for expository texts. Lang. Cogn. Neurosci..

[33] Braga RM, DiNicola LM, Becker HC, Buckner RL (2020). Situating the left-lateralized language network in the broader organization of multiple specialized large-scale distributed networks. J. Neurophysiol..

[34] Hickok G, Poeppel D (2007). The cortical organization of speech processing. Nat. Rev. Neurosci..

[35] Hagoort, P. MUC (Memory, Unification, Control) and beyond. *Front. Psychol*. **4** (2013). 10.3389/fpsyg.2013.00416 PMC3709422 23874313

[36] Duffau H, Moritz-Gasser S, Mandonnet E (2014). A re-examination of neural basis of language processing: Proposal of a dynamic hodotopical model from data provided by brain stimulation mapping during picture naming. Brain Lang..

[37] Blank I, Kanwisher N, Fedorenko E (2014). A functional dissociation between language and multiple-demand systems revealed in patterns of BOLD signal fluctuations. J. Neurophysiol..

[38] Malik-Moraleda S (2022). An investigation across 45 languages and 12 language families reveals a universal language network. Nat. Neurosci..

[39] Hauptman, M., Blank, I. & Fedorenko, E. Non-literal language processing is jointly supported by the language and Theory of Mind networks: Evidence from a novel meta-analytic fMRI approach. (2022). 10.1016/j.cortex.2023.01.013 PMC10210011 37023480

[40] Woolgar A, Duncan J, Manes F, Fedorenko E (2018). Fluid intelligence is supported by the multiple-demand system not the language system. Nat. Hum. Behav..

[41] Schrimpf M (2021). The neural architecture of language: Integrative modeling converges on predictive processing. Proc. Natl. Acad. Sci..

[42] Caucheteux, C. & King, J.-R. *Language processing in brains and deep neural networks: computational convergence and its limits*. 10.1101/2020.07.03.186288 (2020).

[43] Millet, J. *et al*. Toward a realistic model of speech processing in the brain with self-supervised learning. Preprint at http://arxiv.org/abs/2206.01685 (2022).

[44] Toneva, M. & Wehbe, L. Interpreting and improving natural-language processing (in machines) with natural language-processing (in the brain). *ArXiv190511833 Cs Q-Bio* (2019).

[45] Goldstein A (2022). Shared computational principles for language processing in humans and deep language models. Nat. Neurosci..

[46] Jain, S. & Huth, A. G. *Incorporating Context into Language Encoding Models for fMRI*. http://biorxiv.org/lookup/doi/10.1101/327601 (2018).

[47] Markello, R. D. *et al*. *Neuromaps: structural and functional interpretation of brain maps*. http://biorxiv.org/lookup/doi/10.1101/2022.01.06.475081 (2022). 10.1038/s41592-022-01625-w PMC9636018 36203018

[48] Richiardi J (2015). Correlated gene expression supports synchronous activity in brain networks. Science.

[49] Hansen JY (2021). Mapping gene transcription and neurocognition across human neocortex. Nat. Hum. Behav..

[50] *Statistical parametric mapping: the analyis of funtional brain images*. (Elsevier/Academic Press, 2007).

[51] Fischl B, Sereno MI, Tootell RBH, Dale AM (1999). High-resolution intersubject averaging and a coordinate system for the cortical surface. Hum. Brain Mapp..

[52] Gorgolewski, K. J. *et al*. NeuroVault.org: a web-based repository for collecting and sharing unthresholded statistical maps of the human brain. *Front. Neuroinformatics* **9** (2015). 10.3389/fninf.2015.00008 PMC4392315 25914639

[53] ENIGMA. https://enigma.ini.usc.edu/.

[54] EvLab Functional Localization. http://evlab.mit.edu/funcloc/.

[55] Glasser MF (2016). A multi-modal parcellation of human cerebral cortex. Nature.

[56] Oldfield RC (1971). The assessment and analysis of handedness: The Edinburgh inventory. Neuropsychologia.

[57] Diachek E, Blank I, Siegelman M, Affourtit J, Fedorenko E (2020). The Domain-General Multiple Demand (MD) Network Does Not Support Core Aspects of Language Comprehension: A Large-Scale fMRI Investigation. J. Neurosci..

[58] Ivanova AA (2020). Comprehension of computer code relies primarily on domain-general executive brain regions. eLife.

[59] Jouravlev O (2019). Speech-accompanying gestures are not processed by the language-processing mechanisms. Neuropsychologia.

[60] Jouravlev O (2020). Reduced Language Lateralization in Autism and the Broader Autism Phenotype as Assessed with Robust Individual‐Subjects Analyses. Autism Res..

[61] Mollica F (2020). Composition is the Core Driver of the Language-selective Network. Neurobiol. Lang..

[62] Shain, C., Blank, I. A., Fedorenko, E., Gibson, E. & Schuler, W. *Robust effects of working memory demand during naturalistic language comprehension in language-selective cortex*. http://biorxiv.org/lookup/doi/10.1101/2021.09.18.460917 (2021). 10.1523/JNEUROSCI.1894-21.2022 PMC9525168 36002263

[63] Wehbe L (2021). Incremental Language Comprehension Difficulty Predicts Activity in the Language Network but Not the Multiple Demand Network. Cereb. Cortex.

[64] Chen, X. *et al*. *The human language system does not support music processing*. http://biorxiv.org/lookup/doi/10.1101/2021.06.01.446439 (2021).

[65] Scott TL, Gallée J, Fedorenko E (2017). A new fun and robust version of an fMRI localizer for the frontotemporal language system. Cogn. Neurosci..

[66] Thesen S, Heid O, Mueller E, Schad LR (2000). Prospective acquisition correction for head motion with image-based tracking for real-time fMRI. Magn. Reson. Med..

[67] Friston, K. J. *et al*. Spatial registration and normalization of images. *Hum. Brain Mapp*. **3**, 165–189 (1995).

[68] Nieto-Castanon, A. Handbook of functional connectivity magnetic resonance imaging methods in CONN. (Hilbert Press, 2020).

[69] Ashburner, J. & Friston, K. J. Unified segmentation. *NeuroImage* **26**, 839–851 (2005). 10.1016/j.neuroimage.2005.02.018 15955494

[70] Lipkin B, Tuckute G (2022). Open Science Framework.

[71] Fischl B (2008). Cortical Folding Patterns and Predicting Cytoarchitecture. Cereb. Cortex.

[72] Erdoğan, S. B., Tong, Y., Hocke, L. M., Lindsey, K. P. & deB Frederick, B. Correcting for Blood Arrival Time in Global Mean Regression Enhances Functional Connectivity Analysis of Resting State fMRI-BOLD Signals. *Front. Hum. Neurosci*. **10** (2016). 10.3389/fnhum.2016.00311 PMC4923135 27445751

[73] He, H., Shin, D. D. & Liu, T. T. Resting state BOLD fluctuations in large draining veins are highly correlated with the global mean signal. **1** (2010).

[74] Power JD, Schlaggar BL, Petersen SE (2015). Recent progress and outstanding issues in motion correction in resting state fMRI. NeuroImage.

[75] Wong CW, Olafsson V, Tal O, Liu TT (2013). The amplitude of the resting-state fMRI global signal is related to EEG vigilance measures. NeuroImage.

[76] Jouravlev O, Mineroff Z, Blank IA, Fedorenko E (2021). The Small and Efficient Language Network of Polyglots and Hyper-polyglots. Cereb. Cortex.

[77] Murdoch BE (2010). The cerebellum and language: Historical perspective and review. Cortex.

[78] Seydell-Greenwald A, Chambers CE, Ferrara K, Newport EL (2020). What you say versus how you say it: Comparing sentence comprehension and emotional prosody processing using fMRI. NeuroImage.

[79] Kriegeskorte N (2011). Pattern-information analysis: From stimulus decoding to computational-model testing. NeuroImage.

[80] Binder JR, Swanson SJ, Hammeke TA, Sabsevitz DS (2008). A comparison of five fMRI protocols for mapping speech comprehension systems. Epilepsia.

[81] Marek S (2022). Reproducible brain-wide association studies require thousands of individuals. Nature.

[82] Tuckute G (2022). Frontal language areas do not emerge in the absence of temporal language areas: A case study of an individual born without a left temporal lobe. Neuropsychologia.

[83] Lipkin B (2022). figshare.

