# Supplementary material for: Probabilistic atlas for the language network based on precision fMRI data from >800 individuals
Source: Sci Data. 2022 Aug 29;9:529. doi: 10.1038/s41597-022-01645-3 (PMC9424256; doi:10.1038/s41597-022-01645-3)
Supplement: Supplementary file 1 — Supplementary Information [file 41597_2022_1645_MOESM1_ESM.docx]

**Probabilistic atlas for the language network based on precision fMRI data from >800 individuals: Supplementary information**

**Table of Contents**

**SI-1** ……………………………………………………………………………………………… **2**

**SI-2** ……………………………………………………………………………………………… **4**

**SI-3** ……………………………………………………………………………………………… **5**

**SI-4** ……………………………………………………………………………………………… **6**

**SI-5** ……………………………………………………………………………………………… **7**

**SI-6** ……………………………………………………………………………………………… **8**

**SI-7** ……………………………………………………………………………………………… **9**

**SI-1: Comparison of language localization methods.** Response magnitudes for the language and control conditions and the size of the *language > control* effect for three sets of regions of interest (ROIs) in each of the six language parcels (shown on the left, smaller brain in each panel; see Neural Markers) for a (randomly sampled) subset of 403 participants: i) individual functional ROIs (Indiv fROIs), defined as described in Neural Markers; ii) ROIs based on LanA, where within each language parcel (see Neural markers) 10% of voxels with the highest overlap values in LanA are selected based on an independent set of participants (Group LanA; see below for details), and iii) ROIs based on the multi-modal data in the Human Connectome Project created by Glasser et al. (2016) (Glasser Parcels; shown on the right, larger brain in each panel). For the latter, we selected a subset of the parcels (n=23) that had at least 25% voxel overlap with one of the language parcels. Between 2 and 8 parcels overlapped with each of the six language parcels. *A).* Magnitude of the *language* and *control* effects relative to the fixation baseline. Error bars reflect standard error of the mean (SEM) across participants. *B).* Size of the *language > control* effect. For each set of ROIs, each dot corresponds to a language parcel (for Indiv fROIs and Group LanA) or to a Glasser parcel. Vertical black lines mark the average for that set of ROIs. As the figure clearly shows, individually defined fROIs fare best in terms of accurate estimates of effect sizes (and thus remain the gold standard), followed by the Group LanA ROIs, followed by the Glasser parcels. A few of the Glasser parcels in the temporal cortex perform comparably to the Group LanA ROIs, but in the frontal lobe all Glasser parcels grossly underestimate effect sizes (see ^6^ for a discussion of why frontal group-level ROIs are doomed to fail).

Details on the definition of Group LanA ROIs: From the total set of 803 participants for whom we have two or more runs of a language localizer, we took a random sample of 400 participants. The demographic population of this sample was similar to that of the full set (Age = 30.34 ± 7.38 years; ~40% Male, ~60% Female; ~95% Right-handed, ~3 Left-handed, ~2% Ambidextrous; ~79% Native speakers of English, ~21% Proficient speakers of English). From those 400 participants, we generated a probabilistic volumetric atlas as described in Atlas creation. For the remaining 403 participants, we defined ROIs as follows: within each language parcel, we selected—based on the atlas created from the 400 independent participants—the 10% of voxels that have the highest overlap values, i.e., the voxels that are most likely to be language voxels. Critically, unlike for the individual fROIs, the same exact set of voxels is used in every participant, similar to anatomical or multi-modal group ROIs, like the Glasser parcels.

**SI-2: Atlas robustness evaluation.** The robustness of the atlas to variation in data selection and other aspects of the procedure. Each alternative version of the atlas, if evaluating the effect of a sample variable, was developed using the subset of participants adhering to the specified criteria and compared with the atlas based on the non-overlapping subset of participants, or, if evaluating the effect of a scaling or thresholding procedure, was compared with the original atlas (based on the top 10% of *language > control* voxels selected based on the *t-*maps across all 806 participants). For all comparisons, we used a spatial Pearson correlation over the set of all voxels that have non-zero probability values in both versions of the atlas (in order to avoid potentially inflating the correlations). *A).* The atlas generated using only *Localizer Version A* (n=624 participants). Comparison to the atlas generated using *Localizer Versions B-I (n=182 participants)*: *r=*0.890. *B).* The atlas generated using only native English speakers (n=629 participants). Comparison to the atlas generated using only proficient non-native English speakers (n=177): *r=*0.945. *C).* The atlas generated by selecting the top 10% of voxels from the contrast (*con*) maps rather than the variance-normalized *t-*maps. Comparison to LanA: *r=*0.911. *D).* The atlas generated using a fixed *t*-value threshold of 3.09 (~*p*<0.001). Comparison to LanA: *r=*0.957; although, the overlap values are lower: maximum 0.66 in the LH (cf. 0.82 in the original atlas) and maximum 0.40 in the RH (cf. 0.64), since at more stringent *t-*value thresholds, fewer than 10% of voxels across the brain reach significance (e.g., ~3.5% on average at the *p*<0.001 threshold), naturally resulting in lower overlap values relative to the top 10% atlas.

**SI-3: Localizer robustness evaluation.** All 10 localizer versions (Table 2) show strong *language > control* effects. Error bars reflect standard error of the mean (SEM) across participants. For this analysis, we included 803 participants for whom we have at least 2 runs (necessary for across-runs cross-validation, as described in Neural Markers)*.* For each participant, we calculated the average magnitudes across the 6 fROIs of the language-dominant hemisphere, defined as the hemisphere with the larger number of significant voxels (at the *p*<0.001, uncorrected threshold) within the union of the language parcels (see Neural Markers). Across all localizer versions, we see a strong *language > control* effect (in line with past work^10,65^).

**SI-4: Language response stability.** Voxel-level *language > control* effects are stable within individuals across runs. 24 left-lateralized participants were randomly sampled from the set of 803 participants with 2+ runs, and the stability of their spatial topographies over runs was evaluated. For each participant, the subset of voxels contained within the 6 LH language parcels were extracted for both odd and even run splits. Voxel-level BOLD responses were then correlated across run splits both using all voxels, and among only the subset of voxels that present with non-negative effect sizes for both runs (shown in red box in the upper right-hand portion of each plot) so as to ensure that the correlations are not driven by the difference between responsive and non-responsive voxels. The median dominant-hemisphere voxel correlation (across the 803 participants) for the subset of positive-valued voxels across odd and even runs was 0.72 (cf. 0.73 when all voxels were included); and these two sets of voxel correlations were strongly correlated with each other across participants (*r*=0.93), such that a participant who showed a strong correlation for all voxels also showed a strong correlation for the subset of positive-valued voxels. These data confirm that voxel-level effects are highly stable across runs, even when including only responsive voxels.

**SI-5: Atlas sample size convergence.** Atlas topography converges relatively quickly as the number of participants increases. In order to evaluate the stability of the language atlas at various sample sizes, an analysis was conducted whereby non-overlapping subsets of the participants were sampled, atlases derived, and then correlations between those atlases evaluated. Starting from the 806 participants in the dataset, 10 different sample sizes were evaluated: 5, 10, 20, 40, 60, 80, 100, 200, 300, 400. For each sample size (N), 2N participants were sampled from the data, and then split into 2 non-overlapping sets of N participants. Each set was then used to derive a unique volumetric atlas, as described in Atlas Creation, and then the Pearson correlation between the atlases was calculated over the set of voxels that were non-zero in both atlases. This sampling process was repeated for 100 iterations for each N in order to bootstrap a distribution of atlas stability. Each iteration is presented as a point in the figure, with the median and standard deviation overlayed. Atlas stability converges quite quickly, reaching median *r*=0.90 by N=40 (dashed line), and approaching *r*=1.0 at N=400 (dashed line). This information may be helpful for developing probabilistic functional atlases for other networks in the future.

**
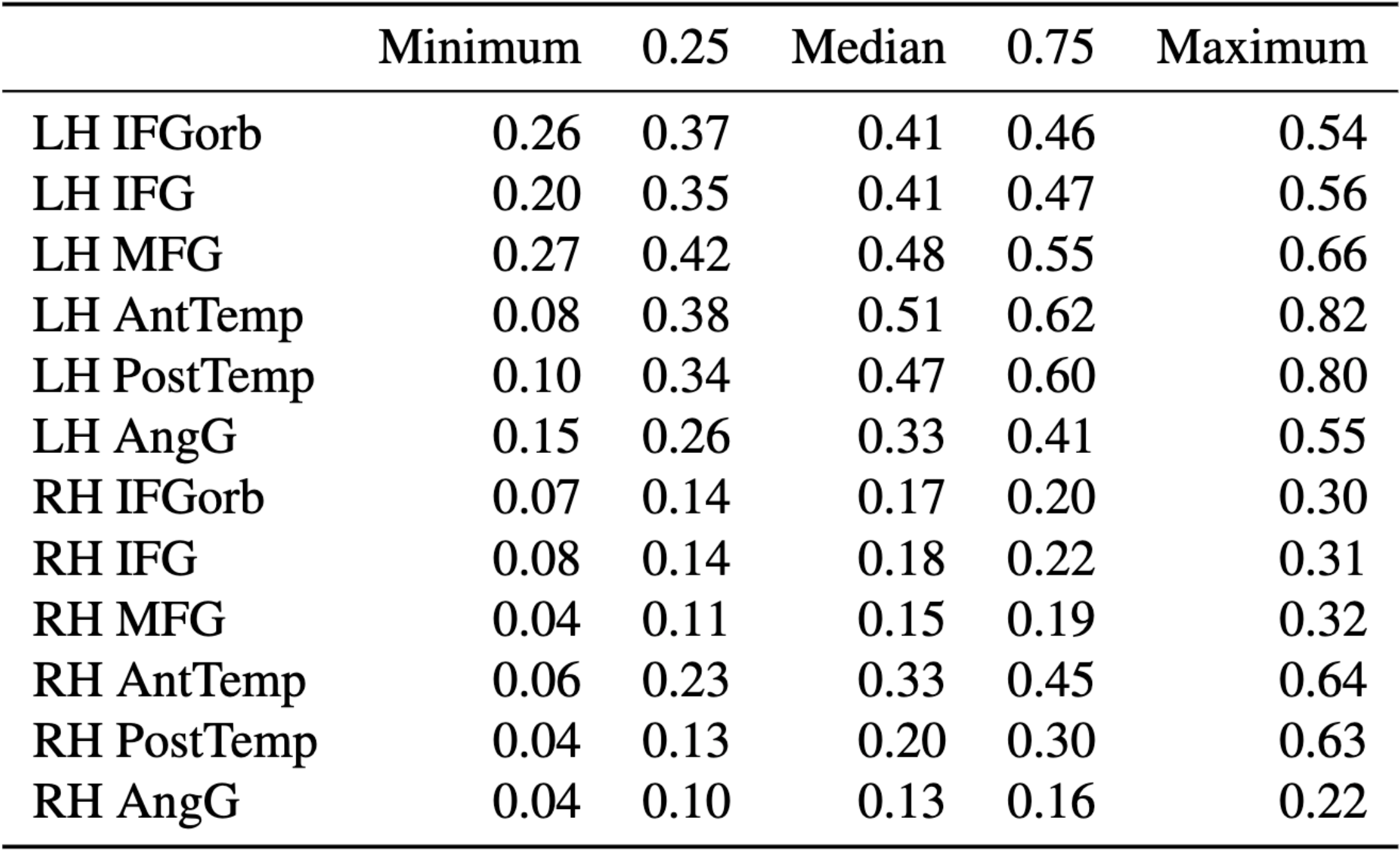
**

**SI-6: ROI-level atlas probabilities.** Minimum, 25^th^ percentile, median, 75^th^ percentile, and maximum probability overlap values within each language parcel (see Neural Markers) of LanA. As expected, LH parcels present with higher probability values than RH parcels. Further, the maximum values are substantially higher in the temporal areas (0.82 and 0.80 in the LH AntTemp and PostTemp parcels, respectively) than in the inferior frontal areas (0.54 and 0.56 in the LH IFGorb and IFG parcels, respectively), in line with much work on the structural and functional variability of the frontal cortex (for a review, see e.g., ^6^).

**SI-7: Random-effects language map.** The probabilistic atlas yields a similar topography to a random-effects group map derived from the same data. The 806 *language > control* maps used to build the atlas were selected for a second-level random-effects analysis, which was run using SPM12. In order to facilitate a comparison between the atlas, which selects for the most responsive voxels, and the random-effects map, which includes both positive- and negative-responding voxels, a mask was defined to select the set of voxels for which both the atlas probability and the random-effects map *t*-statistic were greater than zero. Across the set of 41,147 voxels adhering to this criteria, the probabilistic atlas and random-effects map were found to be highly correlated (*r*=0.91).
